# The Administration of the Synbiotic *Lactobacillus bulgaricus* 6c3 Strain, Inulin and Fructooligosaccharide Decreases the Concentrations of Indoxyl Sulfate and Kidney Damage in a Rat Model

**DOI:** 10.3390/toxins13030192

**Published:** 2021-03-07

**Authors:** Alonso Jerez-Morales, José S. Merino, Sindy T. Díaz-Castillo, Carlos T. Smith, Jorge Fuentealba, Humberto Bernasconi, Gerson Echeverría, Apolinaria García-Cancino

**Affiliations:** 1 Laboratory of Bacterial Pathogenicity, Faculty of Biological Sciences, Universidad de Concepción, 4070386 Concepción, Chile; alojerez@udec.cl (A.J.-M.); csmith@udec.cl (C.T.S.); 2 Pasteur Laboratory, Research and Development Department, 4030635 Concepción, Chile; tatiana.diaz@lpasteur.cl (S.T.D.-C.); hbernasconi@lpasteur.cl (H.B.); 3 Faculty of Veterinary and Agronomy, University of the Americas, 4070254 Concepción, Chile; jmerino@udla.cl; 4 Laboratory of Screening of Neuroactive Compounds, Universidad de Concepción, 4070386 Concepción, Chile; jorgefuentealba@udec.cl; 5 Research Department, Trickster SpA, 8800877 Santiago, Chile; gecheverria@mindsteam.cl

**Keywords:** CKD, indoxyl sulfate, CVD, renal fibrosis, synbiotic

## Abstract

Indoxyl sulfate (IS) is involved in the progression of chronic kidney disease (CKD) and in its cardiovascular complications. One of the approaches proposed to decrease IS is the administration of synbiotics. This work aimed to search for a probiotic strain capable to decrease serum IS levels and mix it with two prebiotics (inulin and fructooligosaccharide (FOS)) to produce a putative synbiotic and test it in a rat CKD model. Two groups of Sprague-Dawley rats were nephrectomized. One group (Lac) received the mixture for 16 weeks in drinking water and the other no (Nef). A control group (C) included sham-nephrectomized rats. Serum creatinine and IS concentrations were measured using high-performance liquid chromatography with diode array detector (HPLC-DAD). Optical microscopy and two-photon excitation microscopy was used to study kidney and heart samples. The Lac group, which received the synbiotic, reduced IS by 0.8% while the Nef group increased it by 38.8%. Histological analysis of kidneys showed that the Lac group increased fibrotic areas by 12% and the Nef group did it by 25%. The synbiotic did not reduce cardiac fibrosis. Therefore, the putative synbiotic showed that function reducing IS and the progression of CKD in a rat model, but no heart protection was observed.

## 1. Introduction

Chronic kidney disease (CKD) is considered a permanent alteration of the kidney structure, its function or, in the most severe cases, both. CKD directly affects the health and life quality of the individual [1,2]. At the physiological level, CKD caused changes frequently observed are increased serum levels of creatinine, cystatin C or ureic nitrogen [2]. The intensified surveillance and the awareness in the population has increased CKD diagnoses reaching, for example, to be determined that a 15% of the USA population is affected by this pathology [3]. The progression of the disease, as well as the effects of the above mentioned metabolites, have a serious impact in multiple systems of the body, including the cardiovascular and the gastrointestinal systems, being the presence of these metabolites associated to an increased mortality in the former and affecting assimilation, motility, and permeability in the latter [4,5]. The gastrointestinal alterations provoke an increased absorption of metabolites in the intestine contributing to sustain a systemic inflammation which, in turn, affects the composition of the normal microbiota and its metabolic functions and may cause intestinal dysbiosis [6]. 

The association between CKD and intestinal dysbiosis might not be explained only by changes in the diet but also, besides, by the increase of the luminal pH, secondary to an increased concentration of ammonium [7]. End-stage CKD patients have marked differences in the abundance of several bacterial taxa in their feces when compared to healthy individuals [8]. Particularly, at the bacterial genus level, CKD patients show an increase of *Enterorhabdus*, *Blautia*, *Ruminococcus*, *Parasutterella*, *Bacteroides*, *Clostridium* (sensu stricto), *Escherichia*, *Shigella,* and *Allobaculum* and a decrease of *Akkermansia*, *Bifidobacterium*, *Clostridium* IV, *Lactobacillus*, *Lactococcus*, *Leuconostoc*, *Alkaliphilus,* and *Pseudomonas* [9,10,11]. Either by the poor renal depuration or by the intestinal dysbiosis, there is a decrease of bacterial genera capable to metabolize and inhibit pathogens which produce toxic metabolites, resulting in their increase [12]. Wong et al. [8] demonstrated that patients suffering advanced CKD showed an increase of bacterial groups possessing enzymes such as urease, uricase and those producing p-cresol and indole.

The accumulation of uremic retention metabolites (URMs) in the blood, including indoxyl sulfate (IS), p-cresyl sulfate (pCS), trimethylamine and trimethylamine N-oxide (TMAO) [5] are postulated as accelerators of CKD [13]. IS, the best-known uremic indole, is secreted by the proximal convoluted tubules of the kidney and it originates from the metabolism of tryptophan by the bacterial tryptophanase. The evidence from animal models shows that IS can affect the renal function and it can damage cells of the tubules generating oxidative stress in endothelial cells, inhibiting repair mechanisms, and favoring interstitial tubular fibrosis because it increases the genic expression of TGF-1, of tissue inhibitor of metalloproteinases (TIMP-1) and of collagen [14]. Nevertheless, the unbalance of the intestinal microbiota, and the concomitant production of toxic metabolites, can be modified. It has been described that the supplementation with certain probiotics and prebiotics may reduce the production of certain uremic toxins and restore the normal microbiota [5]. For example, meta-analysis studies have concluded that CKD patients receiving supplementation with probiotic strains increased their anti-inflammatory cytokines and decreased their levels of uremic toxins [15,16]. Some studies reported that the administration of synbiotics, a combination of probiotics and prebiotics, is capable to decrease the progression of CKD with favorable results [12]. Within the context of the present work, inulin-type fructans are relevant prebiotics because they have shown their capacity to decrease the progression of CKD [17] and to reduce URMs, such as pCS and IS [18]. Panza et al. (2017) administered the probiotics *L. acidophilus* and *Bifidobacterium longum* plus fructooligosaccharides (FOS) in patients with stage 2–5 CKD and reported that untreated patients had significantly more pCS and IS in the blood than patients who received the synbiotic treatment [19]. In this work, we first evaluated the capability of probiotic strains to reduce IS *in vitro* to select the most promising one. Then, this strain was combined with the prebiotics inulin and fructooligosaccharides (FOS) to obtain a putative synbiotic. These prebiotics were selected because they have shown that they can decrease pCS and IS concentration in blood [17,18]. Finally, the efficiency of the putative synbiotic was tested in vivo, evaluating blood IS and creatinine levels and kidney and heart damage using a rat model (Sprague-Dawley rats) including kidney deficient (nephrectomized) and untreated animals. 

## 2. Results

### 2.1. Isolation of Strains Capable to Reduce IS In Vitro

From 36 samples of dairy products, 84 strains were isolated in MRS agar supplemented with bromophenol blue (MRS-BFB), used as a differential medium. This medium allows to differentiate lactic acid bacteria. At pH 3 colonies will be colored yellow and at pH close to 5 they will be colored blue [19]. All the 84 strains isolated were tested in vitro to determine their ability to mediate the reduction of IS. For this, strains were cultured in MRS broth supplemented with 2.8 mM IS during 48 h and then IS was measured by high-performance liquid chromatography (HPLC). Strains 1c2, 6c3, and VIIIc2 were able to significantly reduce (*p* < 0.05) the concentration of the compound (Table 1). Furthermore, the three strains were cultured together for 48 h in the presence of IS to evaluate if they acted synergistically to reduce IS. When all three strains were cultured together in the presence of IS, the concentration of IS was also significantly reduced but less than that achieved by the strains alone, ruling out the possibility of synergistic activity (Table 1).

### 2.2. Identification of the Selected Strains

The three strains selected due to their ability to reduce IS were identified by 16S rRNA sequencing after PCR amplification of DNA. Strain 1c2 was a Gram-positive chain forming coccus which, in the molecular analysis, amplified with the LacZ primers for *Streptococcus thermophilus*. Strains 6c3 and VIIIc2 were both Gram-positive bacilli and amplified with LbG primers to identify genus *Lactobacillus*. The analysis of the 16S rRNA sequencing allowed to identify strain 1c2 as *Streptococcus thermophilus* (97.3% similarity; Access Number: AY188354.1), strain 6c3 as *Lactobacillus bulgaricus* (99.7% similarity; Access Number: CR954253), and strain VIIIc2 as *Lactobacillus acidophilus* (99.5% similarity; Access Number: AY773947.1). Only one of the three strains was selected to prepare the synbiotic and to carry on the following assays. *Streptococcus thermophilus* 1c2 strain was discarded because it showed a low growth rate. From the remaining two strains, *Lactobacillus bulgaricus* 6c3 strain was selected because it showed better probiotic functionality and safety characteristics than *Lactobacillus acidophilus* VIIIc2 strain.

### 2.3. Effect of the Synbiotic in Blood Creatinine and IS Levels

**Figure 1:**
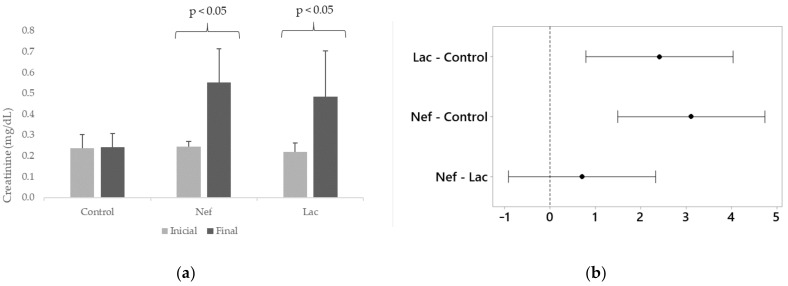
(**a**) Serum creatinine levels of Sprague-Dawley rats at the beginning and at the end of the 16 weeks administration of the synbiotic. (**b**) Differences of the means for final creatinine concentrations with 95% Tukey simultaneous confidence intervals (If an interval does not contain zero, the corresponding means are significantly different). Nef: nephrectomized rats not receiving the synbiotic, Lac: nephrectomized rats receiving the synbiotic, Control: sham-nephrectomized rats.

**Figure 2:**
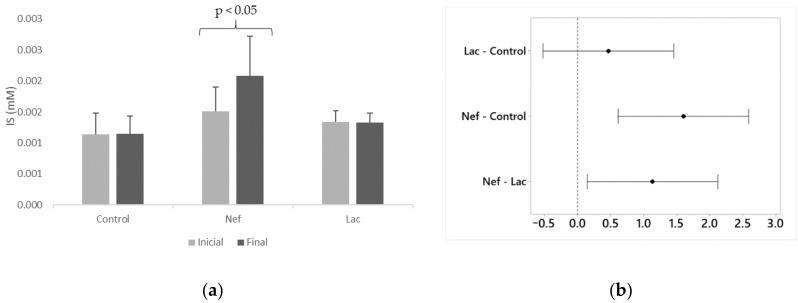
(**a**) Serum indoxyl sulfate (IS) levels of Sprague-Dawley rats at the beginning and at the end of the 16 weeks administration of the synbiotic. (**b**) Differences of the means for final IS concentrations with 95% Tukey simultaneous confidence intervals (If an interval does not contain zero, the corresponding means are significantly different). Nef: nephrectomized rats not receiving the synbiotic, Lac: nephrectomized rats receiving the synbiotic, Control: sham-nephrectomized rats.

**Figure 3:**
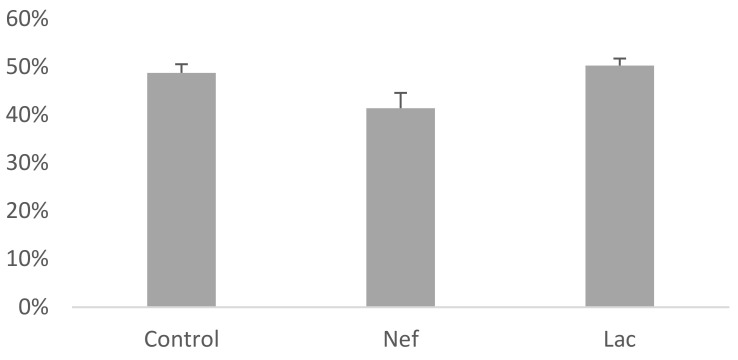
Hematocrit values of Sprague-Dawley rats at the end of the 16 weeks administration of the synbiotic. Nef: nephrectomized rats not receiving the synbiotic, Lac: nephrectomized rats receiving the synbiotic, Control: sham-nephrectomized rats.

The probiotic *L. bulgaricus* 6c3 was lyophilized in association with the prebiotics Inuline and FOS, being the concentration of the probiotic 10^6^ CFU/g of lyophilized product. Ten grams of synbiotic were administered daily, in the drinking water, to the group of nephrectomized synbiotic treated Sprague-Dawley rats (Lac). The nephrectomized untreated group (Nef) and the non-nephrectomized control group did not receive the synbiotic. Serum creatinine and IS levels of the rats were measured by HPLC at the beginning (one week after nephrectomy) and at the end of the assay. Moreover, the hematocrits of the animals of all groups were recorded. The variation of serum creatinine levels at the end of the assay were significantly different (*p* < 0.05) in the Nef and Lac groups, increasing 127% and 121%, respectively, when compared to their initial values (Figure 1). The control group showed a non-significant creatinine increase of only 2%. The increase of creatinine serum levels in the Nef and Lac groups was significantly higher than that of the control group but not significantly different between the Nef and Lac groups. Nevertheless, all three groups maintained their creatinine levels within de normal limits for these rats. On the other hand, serum IS levels significantly increased only in the Nef group corresponding to the nephrectomized (renal deficient) animals not administered the synbiotic (38.8%) (*p* < 0.05). IS levels showed a non-significant 0.5% increase in the control group and a non-significant 0.8% decrease in the Lac group (Figure 2). When the groups of nephrectomized animals were compared, the group not consuming the synbiotic (Nef) showed a significant higher level of IS in comparison with the group who consumed the synbiotic (Lac). There was no significant difference between the Lac group and the control group. The analysis of the data searching for a possible correlation between creatinine and IS levels showed that this correlation was not present (Supplementary Materials: Figures S1–S3). Finally, the results of the hematocrits showed no significant differences among the three groups (Figure 3).

### 2.4. The Histological Analysis Showed that the Administration of the Sybiotic Decreased the Progression of CKD

**Figure 4:**
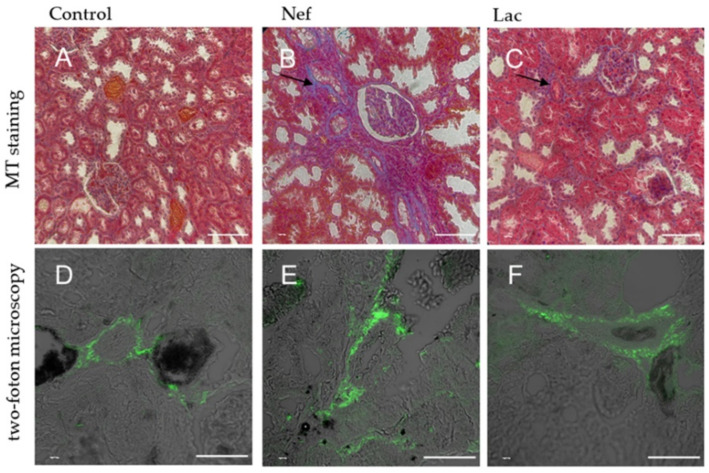
Histological sections of kidneys of Sprague-Dawley rats. (**A**–**C**): Masson’s trichrome stain of nephrons. Fibrotic areas are observed in blue (arrows). (**D**–**F**): same tissue observed by two-photon excitation microscopy. Fibrotic tissue is observed in green. Nef: nephrectomized rats not receiving the synbiotic, Lac: nephrectomized rats receiving the synbiotic, Control: sham-nephrectomized rats. Scale bar: 100 µm for (**A**–**C**) micrographs and 50 µm for (**D**–**F**) micrographs.

At the end of the experiment, rats were euthanized to obtain samples from the left kidneys and hearts to be processed for histological analysis. The fibrotic area was determined by the Masson’s trichrome (MT) stain using the informatic tool Image-Pro Plus 3.0 (Media Cybernetics, Rockville, MD, USA) to calculate the percentage of damaged (i.e., fibrotic) area (Table 2) and by two-photon excitation microscopy, a method that detects collagen by the emission of two-photons, causing the collagen to generate a second harmonic, which can be visualized by confocal microscopy getting a specific image close to the organization and distribution of collagen inside of the tissue. When comparing the fibrotic areas of the kidneys, evaluated by the MT stain, it was possible to observe that fibrotic areas, were 25% larger in the Nef group but only 12% larger in the Lac group, administered the synbiotic when compared to sham-nephrectomized control animals. Lesions observed with this staining procedure were located at the parietal layer of the Bowman’s capsule and the basement membrane of the proximal and distal convoluted tubules of the animals of groups Nef and Lac. In the case of the Nef group, including the animals not administered the synbiotic, a loss of cytoplasm of the cells of the convoluted tubules was observed with concomitant development of fibrotic tissue. When the tissues of the kidneys were analyzed by two-photon excitation microscopy, the collagen was observed in all samples having a mesh type conformation. Groups Nef and Lac showed more lesions than the control group, in which only one fibrotic area was found (Figure 4). No lesions were found in the renal corpuscles in any sample when observed under two-photon excitation microscopy.

### 2.5. The Administration of the Synbiotic Did Not Attenuate Cardic Fibrosis

**Figure 5:**
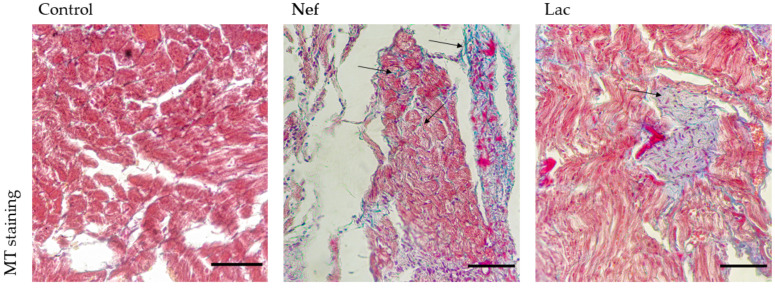
Masson’s trichrome (MT) staining of the heart of Sprague-Dawley rats. Fibrotic areas are stained in blue (arrows). Nef: nephrectomized rats not receiving the synbiotic, Lac: nephrectomized rats receiving the synbiotic. Control: sham-nephrectomized rats. Scale bar 100 µm.

In the heart, the MT stain showed the presence of fibrotic areas in the tissue of nephrectomized rats, while these areas were absence in the control animals. The induction of CKD in rats generates cardiac fibrosis which was not prevented by the administration of the synbiotic treatment in nephrectomized rats (Figure 5).

## 3. Discussion

Considering its significant increase in worldwide prevalence, the difficulties and cost of treatment, and because it is one of the main risk factors for cardiovascular disease [13,20], CKD is becoming one of the main public health challenges. Presently, scientific evidence supporting a close relationship between intestinal health and renal health, commonly referred as intestinal-renal axis, is growing [21]. The communication/interaction between the intestinal microbiota and its host is patho-physiologically relevant, particularly in CKD patients [22]. In these patients, the increase of uremic compounds in the blood may affect the composition and metabolism of the microbiota and also favor the growth of bacterial groups which produce these metabolites [21,22]. In fact, it has been reported that, in individuals suffering dysbiosis, the microbiota can produce over 200 serum metabolites [17]. All the above implies an increase of intestinal permeability which may result in translocation of metabolites, endotoxins, and viable bacteria into the bloodstream [23], thus favoring chronic inflammation, increasing cardiovascular risk, and worsening uremic toxicity. Nevertheless, the intestinal microbiota unbalance may be reverted, for example, using prebiotics, probiotics, or their combination as synbiotics. In this context, it has been described that supplementation with certain probiotics and prebiotics might reduce the production of certain uremic toxins and restore normal microbiota [5]. Our results provide evidence that, from the 84 bacterial strains tested in vitro in this study, three strains showed the ability to significantly reduce IS in the culture medium. These results are similar with those reported by other authors [24,25]. The mechanism by which IS is reduced remains uncertain yet. It has been postulated that IS may bind to the cell wall of probiotics and then be eliminated with feces [25] or that some microorganisms possess enzymes capable to degrade IS [26]. Whether these postulated mechanisms are correct or not, it has been widely reported that probiotics restore the normal microbiota in individuals having CKD caused dysbiosis [27].

The use of prebiotics and probiotics as an approach to decrease the progression of CKD has been extensively investigated. Various prebiotics have demonstrated to be useful to reduce different dysbiosis metabolites, as peritoneal dialysis supplement [17,18]. Lai et al. [28] compared the effect of a low protein diet and the supplementation of this diet with inulin (19 g/day) in stage 3 or 4 CKD patients. They reported that the dietary intervention had little effect in the microbiota, only reducing the frequency of genus *Lactobacillus*, but when inulin was added it was possible to increase the levels of *Bifidobacterium*. Another beneficial effect of inulin was a reduction of intestinal pH, of ureic nitrogen, of TNF-a and NOX2 in blood, limiting the increase of URMs and reducing inflammation [28]. Nevertheless, it has been reported that the addition of probiotics to the diet generates much more significant effects, leading to the use of prebiotics and probiotics mixtures (synbiotics) to potentiate their microbiota modulating activity. Alla and Sadeek [29] evaluated the administration of Arabic gum and *L. casei* Shirota to Wistar rats suffering induced CKD. Their results showed that the treatment was able to reduce blood concentrations of urea, creatinine, and uric acid. Similarly, it has been extensively reported that the use of prebiotics, probiotics, and synbiotics possess a modulating effect on the intestinal microbiota. This is an important issue because, under dysbiosis, uremic toxins (particularly indole derivatives) have shown to activate the pregnane X receptor (PXR) which regulates the expression of TLR4. The resulting increased inflammation affects the intestinal barrier augmenting its permeability to various metabolites and allowing the translocation of bacteria [11]. In the present work, the treatment consisted in the administration of a synbiotic including inulin, FOS and *L. bulgaricus* 6c3 strain. Rocchetti et al. [30] demonstrated that a different *L. bulgaricus* strain, combined in a synbiotic, decreased IS and PCS serum levels, when used associated to an innovative dialysis treatment in HD patients.

Differences in the serum creatinine levels were observed between the control and nephrectomized (Lac and Nef) rats indicating that the uremic model was successfully established. Nevertheless, no significant difference was observed when comparing groups Lac and Nef. That is to say, there was no difference in creatinine levels if the treatment was administered or not to CKD rats, an observation consistent with other reports [24,31,32]. Thus, the contribution of creatinine measurements was to confirm the kidney deficiency achieved by nephrectomy. On the other hand, serum IS levels increased significantly in the Nef group (38.8%) but not in the synbiotic administered Lac group whose IS level was similar to that of the control group. Thus, the synbiotic was able to avoid the damaging increase of IS concentrations despite the presence of induced CKD. Several works have reported similar results when administering prebiotics [28], probiotics [24] or synbiotics [29] because they have the capacity to decrease IS in different models of study. Wu et al. [31] administered *L. acidophilus* LB to 5/6th nephrectomized Sprague-Dawley rats and evaluated 35 metabolites in feces. Interestingly, some metabolites (3-(3-hydroxyphenyl) propionic acid, amebamide, benzopyrene, aspartyl-glutamine, phenethylamine glucuronide, and T2 toxin tetrol) increased five times in nephrectomized rats; nevertheless, their levels were restored to normal levels after treatment. These results are in agreement with our observations because the administration of the synbiotic maintained IS levels similar that of the control group.

It has been reported that IS induces a local increase of reactive oxygen species (ROS) in the renal tissue which, in turn, activate the nuclear factor KappaB (NF-kB) and activate the oxidative stress and the production of pro-inflammatory cytokines [11]. IS is capable to enter into cells of convoluted tubules using the transporter for organic anions and, once there, it increases ROS and NF-kB, leading to a decrease of E-cadherin and an increase of the transforming growth factor β (TGF-β) and smooth muscle actin alfa (α-SMA). The result is an epithelial mesenchymatic transition producing renal fibrosis [33]. Similarly, one of the main kidney protecting mechanisms of *L. bulgaricus* 6c3 is its anti-inflammatory activity achieved decreasing IS levels. Our observations showed that the kidney fibrotic area in animals of the nephrectomized Nef group (25% of fibrotic area) was twice as large than that of synbiotic administered nephrectomized Lac rats (12% of fibrotic area) and sham-nephrectomized control rats; thus, the treatment with the synbiotic was effective to reduce the fibrotic area of the kidney in CKD rats. Theoretically, this protecting effect can be explained by an alteration of the intestinal lumen which, in turn, could reduce IS serum concentrations. Feng et al. [34] demonstrated significant changes between nephrectomized and non-nephrectomized rats involving 13 bacterial species belonging to genera *Blautia*, *Escherichia_Shigella*, *Bacteroides*, *Allobaculum Clostridium*_IV and of 291 metabolites; thus, emphasizing the impact of CKD on the microbiota and URMs. Moreover, they also provided evidence that the administration of two prebiotics (*Poria cocos* and poricoic acid A) modified the intestinal lumen and restored the intestinal normal microbiota. Both prebiotics mitigated inflammation and oxidative stress through the inhibition of the IκBα /NF-κB I pathway and normalized serum urea and creatinine as well as α-SMA expression. So far, to the best of our knowledge, this is the first work reporting the use of two-photon excitation microscopy in this type of studies and, interestingly, although there is evidence of damage in the glomerular corpuscle (Masson trichrome stain), the fibrotic lesions were only observed in the convoluted tubules by two-photon excitation microscopy. This observation supports those reports mentioning that the main deleterious effect of the most studied URMs is, precisely, on the cells of the convoluted tubules of the kidney [33].

It has been reported that IS induces cardiac fibrosis with the expression of fibrotic proteins (TGF- β1, SMA and type 1 collagen), cardiac hypertrophy and oxidative stress [35] and an increasing number of publications is confirming the negative effects of IS on the cardiovascular system. Kamiński et al. [36] studied 51 patients in CKD stages 1 to 5 and reported that the accumulation of IS was correlated with disturbances of some factors affecting parameters of the hemostatic system of CKD patients, such as H_2_O_2_ and plasma Cu/Zn superoxide dismutase. It has been also reported that the administration of a probiotic strain can reduce the biomarkers of inflammation in cardiovascular disease and to reduce its associated risks [37,38]. Our results indicated that, although serum IS was significantly reduced, the administration of the synbiotic did not avoid the development of cardiac fibrosis. In fact, it has been postulated that, despite the damage produced by IS in the cardiovascular system, the main URMs affecting the heart are p-cresol [39] and TMAO [40], metabolites not evaluated in this study. Nevertheless, some authors state that differences among the results of different studies are the consequence of the variability of the study models. Karbowska and coworkers [41], working with Wistar rats and C57BL6/cmdb mice, reported the effect of acute exposure to IS on the thrombotic process, clotting, coagulation and erythrocyte osmotic resistance among other parameters. They reported that 0.1 mM IS in blood was correlated with fibrin generation and collagen induced aggregation in PRP and that up to 1 mM plasmatic IS had no effect on erythrocyte hemolysis. In our work, using Sprague-Dawley rats, the highest IS levels reached were nearly 0.0025 mM in the nephrectomized group not consuming the synbiotic (Nef). It is possible that IS levels above 0.0025 mM might have revealed a correlation between creati-nine and IS levels. Regarding our results on the hematocrit of all three animal groups, they are consistent with the report of Karbowska and coworkers. They also concluded that IS may be one crucial factor of heart damage. Since we also observed heart damage with lower IS levels, perhaps our model using nephrectomized Sprague-Dawley rats might contribute to detect other key factors causing heart damage. Finally, it must be emphasized that the higher levels of IS reported by Karbowswka and coworkers when compared to those reported in the present work can probably be the consequence that they injected up to 100 mg/kg of body weight of IS to the animals while our IS levels were the consequence of 5/6th nephrectomy of them. Although the increase of publications pertaining the administration of probiotics in cardiac and other diseases there is still a long path to walk before comprehending how prebiotics and probiotics contribute to our health.

## 4. Conclusions

The results here reported demonstrate that the synbiotic formulated using the strain *L. bulgaricus* 6c3, inulin, and FOS is capable to reduce IS both in vitro and in the blood of Sprague-Dawley rats used model. Moreover, it was able to reduce the progression of renal fibrosis when administered daily but it was unable to avoid the development of cardiac fibrosis.

## 5. Materials and Methods

### 5.1. Bacterial Isolation

Thirty-six dairy products were obtained at the local market and bacteria isolated at the Laboratory of Bacterial Pathogenicity, Department of Microbiology, University of Concepción, Chile. Samples of each product (1 g or 1 mL) were suspended in 9 mL De Man, Rogosa and Sharpe (MRS) broth (Becton Dickinson, BD, Franklin Lakes, NJ, USA) and incubated during 24–48 h under microaerobic conditions. Then, aliquots were seeded in plates containing MRS agar (Becton Dickinson, BD) supplemented with bromophenol blue (MRS-BPB) [19]. For this, 70 g MRS were dissolved in 1 L distilled water and 0.05% l-cysteine-HCL (Sigma-Aldrich, St. Louis, MO, USA) was added. The solution was heated, agitated until dissolved, 0.002% BPB (*w*/*v*) was added and then autoclaved. After the medium was plated, samples were seeded and incubated at 37 °C, during 24–48 h under microaerobic conditions. After incubation, the isolated colonies were incubated in MRS broth for 24–48 h at 37 °C and stored in vials with 20% glycerol at −80 °C.

### 5.2. Determintion, by HPLC-DAD, of Strains Capable to Reduce IS

To evaluate their in vitro ability to reduce the concentration of IS, the strains were incubated in MRS broth supplemented with 2.8 mM (600 mg/L) IS (hereafter referred as MRS-IS). For this, 50 mL of MRS-IS broth were inoculated with each strain (5% *v*/*v*) and cultured for 48 h under microaerophilic condition. Before culturing, a 0.5 mL aliquot was obtained (initial time) and another aliquot was obtained at the end of culturing (final time). Samples were centrifuged (13,000× *g*, 5 min) and, after been filtered using 0.22 μm sterile membranes, were assayed by HPLC. 

IS concentrations were quantified in the supernatants using an HPLC equipment fitted with a DAD detector (Elite LaChrom L-2455) and a C18 column of 5 µm (4.6 × 250 mm) (InertSustain, Torrance, CA, USA) kept at 30 °C. The mobile phase consisted of a concentration gradient including acetonitrile, methanol, and water (26.3:15:58.7) for 13 min, then the mobile phase was replaced by another one including acetonitrile and 1% phosphoric acid (3:17) for 47 min. Flow was set at 0.7 mL/min. Absorbance was measured at 210 nm at 30 °C.

### 5.3. DNA Amplification and Identification of the Strains

For this purpose, 12.5 μL of the commercial kit SapphireAmp Fast PCR Master (MixGoTaq), 1.5 μL of oligonucleotides LbG-F (5’-AGAAGAGGACAGTGGAAC-3’) and LbG-R (5’-TTACAAACTCTCATGGTGTG-3’) for *Lactobacillus* characterization and LacZ-F (5’-CACTATGCTCAGAATACA-3’) and LacZ-R (5’-CGAACAGCATTGATGTTA-3’) for *Streptococcus thermophilus* characterization, 5 μL of bacterial DNA template of each strain and 5 μL nucleases-free water (IDTDNA, Coralville, IA, USA) were used. Amplification was performed using a BIOER XP Cycler (Bioer, Hangzhou, China) thermocycler and it consisted of an initial denaturation at 94 °C during 1 min followed by 30 cycles including denaturation at 98 °C during 30 s, annealing during 30 s at 58 °C for LbG and 54 °C for LacZ and extension at 72 °C during 40 s. A final extension was carried out at 72 °C during 8 min. PCR products were analyzed after electrophoresis in 1.2% (*w*/*v*) agarose gels (CleaverGEL, Warwickshire, UK, CSL-100), at 110 V for 90 min in an horizontal electrophoresis chamber (ENDURO, Houston, TX, USA, 7.10 Horizontal Gel Box) connected to a 300 V power source (Nyxtechnik, San Diego, CA, USA, Voltronyx, Reactor 37). Agarose gels were stained using GelRed (Biotium, Hayward, CA, USA) and visualized using an UV system for molecular images registering (ENDURO, GDS Gel Documentation System). Furthermore, the sequencing of 16S rRNA was done with the primers 18-F (5’-CACCAGGTTGATTCTGCC-3’) and 1492-R (5´-GGTTACCTTGTTACGACTT-3´) following the previous steps (but 56 °C as annealing temperature). The amplicons were sequenced and analyzed by Macrogen (Seoul, Korea). All amplifications were done in duplicate, using DNases-free water as negative control.

### 5.4. Effect of the Administration of the Synbiotic to Nephrectomized Sprague-Dawley Rats

This assay was performed using 12 weeks old female Sprague-Dawley rats. Rearing of animals and their experimental use were done in accordance with national regulations and all studies were approved by the Committee of Bioethics and Biosafety, Faculty of Biological Sciences, University of Concepcion. Rats were feed ad libitum in a room kept constantly at 25 °C and with a 12/12 photoperiod. CKD was induced by reduction of the renal mass (5/6th) by nephrectomy after anesthetizing the animals using Xylazine (Drag Pharma, Santiago, Chile) (6 µg/g weight) and Ketamine (Drag Pharma) (90 µg/g weight). Nephrectomy was carried out as described by Wang et al. [42]. The right kidney was extracted and both poles of the left were ligated to reach a 75% reduction of the total renal mass. Fifteen rats were nephrectomized while control animals were subjected to surgical stress. After surgery, animals received a maintenance diet. Three groups of animals were considered in the study: one group was the control group (C) (*n* = 5), a second group included nephrectomized untreated animals (Nef) (*n* = 5) and the third group included nephrectomized animals treated with the synbiotic (Lac) (*n* = 5). Synbiotic treatment (10^7^ CFU) was administered daily in the drinking water, for 16 weeks, after the first week of surgery. Rats of the three groups (C, Nef and Lac) were sacrificed 17 weeks after surgery using the accepted euthanasia method for laboratory rats [43] involving Xylazine/Ketamine induced anesthesia and cervical dislocation. Blood samples were obtained at the beginning of the treatment (one week after inducing CKD) and at the end of the experiment (week 17) for assaying URM and to evaluate hematocrit. Further, kidneys and hearts were removed for their histological analysis.

To obtain the synbiotic, 4 L of MRS broth were inoculated with *L. bulgaricus* 6c3 strain and incubated for 24 h in a thermoshaker (Velp scientifica, Via Stazione, Italy) and then a pellet obtained by centrifugation (Gyrozen, Gimpo, Korea) at 3000× *g* for 5 min. The pellet was suspended in 500 mL of sterilized water and centrifuged under the same conditions previously described. The pellet was resuspended in 500 mL of a matrix of sterilized water containing 4% inulin and 4% fructooligosaccharide (FOS) and frozen at −50 °C in flasks suitable for lyophilization. Lyophilization was carried out at 10 psi for 36 h (Biobase, Shandong, China). After lyophilization, the synbiotic was aliquoted and each aliquot was kept under vacuum at 4 °C until use. Regarding its stability, the synbiotic was monitored during the 16 weeks of the experiments and even after that for up to one year showing no signs of deterioration. In order to adjust the synbiotic to 10^7^ cfu of drinking water, a sample of lyophilized *L. bulgaricus* 6c3 was plated in MRS agar to determine its viable count.

### 5.5. Assesment of Creatinine and IS Concentration

Blood samples were obtained and URMs were extracted from the serum using the Ostro kit (Waters, Milford, MA, USA). All samples were evaluated by means of an HPLC equipment furnished with a DAD detector (Elite LaChrom L-2455) using a reverse phase column C18 of 5 µm (4.6 × 250 mm) (InertSustain). The mobile phase, times and flows were the same as those reported in Section 5.2. Absorbance was measured at 205 nm for creatinine and 220 nm for IS.

### 5.6. Histological Analysis

Kidney and heart samples were fixed using 4% paraformaldehyde and processed until embedding in paraffin wax. Sections were obtained using a microtome set at a thickness of 7 μm. Finally, sections were stained using Masson’s trichrome stain (Diapath, Martinengo, Italy). Histological sections were observed under an optical microscope. The percentages of fibrotic areas were determined using a semiquantitative analysis following evaluation of the fibrotic and non-fibrotic areas of 10 microscopic fields using the Image-Pro Plus 3.0 (Media Cybernetics) software. After evaluating the damage, collagen was characterized by the previously sectioned samples using two-photon excitation microscopy (LSM780 Zeiss, Jena, Germany) following the same procedure of Muñoz and coworkers [44], except that a pulsed IR laser at 760 nm was used instead of 790 nm used by Muñoz and coworkers. 

### 5.7. Statistical Analysis

In order to compare the results of the initial evaluation of the numerical variables, an analysis of variance (one way ANOVA) was performed, and the Tukey’s multiple comparison test was used when the result of the ANOVA was significant (Minitab, Coventry, UK). To be considered as statistically significant, the *p* values associated to the assays required to be equal or lesser than 0.05.

## Figures and Tables

**Table 1:** In vitro decrease if indoxyl sulfate (IS), evaluated by high-performance liquid chromatography (HPLC), mediated by bacterial strains.

Strains	Time 0 h (mM)	Time 48 h (mM)	Decrease (%)
1c2	2.77 ± 0.13	2.48 ± 0.05	11%
6c3	2.93 ± 0.23	2.47 ± 0.07	16%
VIIIc2	3.49 ± 0.10	3.04 ± 0.02	12%
1c2 + 6c3 + VIIIc2	2.71 ± 0.01	2.53 ± 0.09	7%

Three of 84 strains tested were able to mediate a significant decrease of IS.

**Table 2:** Percentage of fibrotic area in Masson’s trichrome stained kidney histological sections. Results are expressed as mean ± standard error.

Group	Fibrotic Area (%)
Control	0 ± 0
Nef	25 ± 1.9
Lac	12 ± 1.23

## Data Availability

Not applicable.

## Supplementary Materials

The following are available online at https://www.mdpi.com/2072-6651/13/3/192/s1. Figure S1: Correlation between creatinine and IS levels of the control group, Figure S2: Correlation between creatinine and IS levels of the Nef group and Figure S3: Correlation between creatinine and IS levels of the Lac group.

## References

[1] Zoccali C., Vanholder R., Massy Z.A., Ortiz A., Sarafidis P., Dekker F.W., Fliser D., Fouque D., Heine G.H., Jager K.J. (2017). Cardiovascular Medicine Working Group of the European Renal Association—European Dialysis Transplantation, A. The systemic nature of CKD. Nat. Rev. Nephrol..

[2] Romagnani P., Remuzzi G., Glassock R., Levin A., Jager K.J., Tonelli M., Massy Z., Wanner C., Anders H.J. (2017). Chronic kidney disease. Nat. Rev. Dis. Primers..

[3] CDC (2019). Chronic Kidney Disease in the United States. Availabeonline:https://www.cdc.gov/kidneydisease/pdf/2019_National-Chronic-Kidney-Disease-Fact-Sheet.pdf.

[4] Moradi H., Sica D.A., Kalantar-Zadeh K. (2013). Cardiovascular burden associated with uremic toxins in patients with chronic kidney disease. Am. J. Nephrol..

[5] Meijers B., Evenepoel P., Anders H.J. (2019). Intestinal microbiome and fitness in kidney disease. Nat. Rev. Nephrol..

[6] Carding S., Verbeke K., Vipond D.T., Corfe B.M., Owen L.J. (2015). Dysbiosis of the gut microbiota in disease. Microb. Ecol. Health Dis..

[7] Vaziri N.D., Wong J., Pahl M., Piceno Y.M., Yuan J., DeSantis T.Z., Ni Z., Nguyen T.H., Andersen G.L. (2013). Chronic kidney disease alters intestinal microbial flora. Kidney Int..

[8] Wong J., Piceno Y.M., DeSantis T.Z., Pahl M., Andersen G.L., Vaziri N.D. (2014). Expansion of urease- and uricase-containing, indole- and p-cresol-forming and contraction of short-chain fatty acid-producing intestinal microbiota in ESRD. Am. J. Nephrol..

[9] Kieffer D.A., Piccolo B.D., Vaziri N.D., Liu S., Lau W.L., Khazaeli M., Nazertehrani S., Moore M.E., Marco M.L., Martin R.J. (2016). Resistant starch alters gut microbiome and metabolomic profiles concurrent with amelioration of chronic kidney disease in rats. Am. J. Physiol. Renal Physiol..

[10] Cani P.D. (2019). Microbiota and metabolites in metabolic diseases. Nat. Rev. Endocrinol..

[11] Yoshifuji A., Wakino S., Irie J., Matsui A., Hasegawa K., Tokuyama H., Hayashi K., Itoh H. (2018). Oral adsorbent AST-120 ameliorates gut environment and protects against the progression of renal impairment in CKD rats. Clin. Exp. Nephrol..

[12] Ranganathan N., Vyas U., Hanlon K., Ranganathan P., Irvin A. (2018). Improvements in Glomerular Filtration Rate (GFR) in Chronic Kidney Disease (CKD) Patients Using a Commercial Patented and Proprietary Probiotic Prebiotic Formulation* -3rd Biennial Survey. Int. J. Nephrol. Kidney Fail.

[13] Tao S., Tao S., Cheng Y., Liu J., Ma L., Fu P. (2019). Effects of probiotic supplements on the progression of chronic kidney disease: A meta-analysis. Nephrology.

[14] Sircana A., De Michieli F., Parente R., Framarin L., Leone N., Berrutti M., Paschetta E., Bongiovanni D., Musso G. (2019). Gut microbiota, hypertension and chronic kidney disease: Recent advances. Pharmacol. Res..

[15] Jia L., Jia Q., Yang J., Jia R., Zhang H. (2018). Efficacy of Probiotics Supplementation on Chronic Kidney Disease: A Systematic Review and Meta-Analysis. Kidney Blood Press. Res..

[16] Jacouton E., Michel M.-L., Torres-Maravilla E., Chain F., Langella P., Bermúdez-Humarán L.G. (2019). Elucidating the Immune-Related Mechanisms by Which Probiotic Strain *Lactobacillus casei* BL23 Displays Anti-tumoral Properties. Front. Microbiol..

[17] Li L., Xiong Q., Zhao J., Lin X., He S., Wu N., Yao Y., Liang W., Zuo X., Ying C. (2020). Inulin-type fructan intervention restricts the increase in gut microbiome–generated indole in patients with peritoneal dialysis: A randomized crossover study. Am. J. Clin. Nutr..

[18] Panza F., Duranti D., Chiara R., Basile M., Bagnati M.J.A.R.D.M. (2017). Short-Term Effects of Pre/Probiotics on P-Cresol and Indoxyl-Sulphate Serum Concentrations During the Various Stages of Chronic Kidney Disease. Arch. Renal. Dis. Manag..

[19] Lee H.M., Lee Y. (2008). A differential medium for lactic acid-producing bacteria in a mixed culture. Lett. Appl. Microbiol..

[20] Koppe L., Mafra D., Fouque D. (2015). Probiotics and chronic kidney disease. Kidney Int..

[21] Sabatino A., Regolisti G., Brusasco I., Cabassi A., Morabito S., Fiaccadori E. (2015). Alterations of intestinal barrier and microbiota in chronic kidney disease. Nephrol. Dial. Transplant..

[22] Poesen R., Evenepoel P., de Loor H., Kuypers D., Augustijns P., Meijers B. (2016). Metabolism, Protein Binding, and Renal Clearance of Microbiota–Derived p-Cresol in Patients with CKD. Clin. J. Am. Soc. Nephrol..

[23] Anders H.J., Andersen K., Stecher B. (2013). The intestinal microbiota, a leaky gut, and abnormal immunity in kidney disease. Kidney Int..

[24] Fang C.Y., Lu J.R., Chen B.J., Wu C., Chen Y.P., Chen M.J. (2014). Selection of uremic toxin-reducing probiotics in vitro and in vivo. J. Funct. Foods.

[25] Nowak A., Libudzisz Z. (2007). Ability of intestinal lactic bacteria to bind or/and metabolise phenol and p-cresol. Ann. Microbiol..

[26] Wang F., Jiang Y.S., Liu F. (2016). The influence of mutant lactobacilli on serum creatinine and urea nitrogen concentrations and renal pathology in 5/6 nephrectomized rats. Ren. Fail..

[27] Lee Y.J., Li K.Y., Wang P.J., Huang H.W., Chen M.J. (2020). Alleviating chronic kidney disease progression through modulating the critical genus of gut microbiota in a cisplatin-induced Lanyu pig model. J. Food Drug Anal..

[28] Lai S., Molfino A., Testorio M., Perrotta A.M., Currado A., Pintus G., Pietrucci D., Unida V., La Rocca D., Biocca S. (2019). Effect of Low-Protein Diet and Inulin on Microbiota and Clinical Parameters in Patients with Chronic Kidney Disease. Nutrients.

[29] Alla F., Sadeek E.A. (2018). Effect of Arabic Gum as prebiotics and *Lactobacillus casei* Shirota (LcS) as probiotic on oxidative stress and renal function in adenine–induced chronic renal failure in rats. Eur. J. Nutr. Food Saf..

[30] Rocchetti M.T., Cosola C., di Bari I., Magnani S., Galleggiante V., Scandiffio L., Dalfino G., Netti G.S., Atti M., Corciulo R. (2020). Efficacy of Divinylbenzenic Resin in Removing Indoxyl Sulfate and P-Cresol Sulfate in Hemodialysis Patients: Results from an In Vitro Study and an In Vivo Pilot Trial (xuanro4-Nature 3.2). Toxins.

[31] Wu B., Jiang H., He Q., Wang M., Xue J., Liu H., Shi K., Wei M., Liang S., Zhang L. (2017). Liquid Chromatography/Mass Spectrometry Reveals the Effect of *Lactobacillus* Treatment on the Faecal Metabolite Profile of Rats with Chronic Renal Failure. Nephron.

[32] Wanchai K., Yasom S., Tunapong W., Chunchai T., Eaimworawuthikul S., Thiennimitr P., Chaiyasut C., Pongchaidecha A., Chatsudthipong V., Chattipakorn S. (2018). Probiotic *Lactobacillus paracasei* HII01 protects rats against obese-insulin resistance-induced kidney injury and impaired renal organic anion transporter 3 function. Clin. Sci..

[33] Vanholder R., Schepers E., Pletinck A., Nagler E.V., Glorieux G. (2014). The uremic toxicity of indoxyl sulfate and p-cresyl sulfate: A systematic review. J. Am. Soc. Nephrol..

[34] Feng Y.L., Cao G., Chen D.Q., Vaziri N.D., Chen L., Zhang J., Wang M., Guo Y., Zhao Y.Y. (2019). Microbiome-metabolomics reveals gut microbiota associated with glycine-conjugated metabolites and polyamine metabolism in chronic kidney disease. Cell. Mol. Life Sci..

[35] Yisireyili M., Shimizu H., Saito S., Enomoto A., Nishijima F., Niwa T. (2013). Indoxyl sulfate promotes cardiac fibrosis with enhanced oxidative stress in hypertensive rats. Life Sci..

[36] Kamiński T.W., Pawlak K., Karbowska M., Myśliwiec M., Pawlak D. (2017). Indoxyl sulfate—The uremic toxin linking hemostatic system disturbances with the prevalence of cardiovascular disease in patients with chronic kidney disease. BMC Nephrol..

[37] Liu D.-M., Guo J., Zeng X.-A., Sun D.-W., Brennan C.S., Zhou Q.-X., Zhou J.-S. (2017). The probiotic role of *Lactobacillus plantarum* in reducing risks associated with cardiovascular disease. Int. J. Food Sci. Technol..

[38] Malik M., Suboc T.M., Tyagi S., Salzman N., Wang J., Ying R., Tanner M.J., Kakarla M., Baker J.E., Widlansky M.E. (2018). *Lactobacillus plantarum* 299v Supplementation Improves Vascular Endothelial Function and Reduces Inflammatory Biomarkers in Men with Stable Coronary Artery Disease. Circ. Res..

[39] Lin C.J., Wu V., Wu P.C., Wu C.J. (2015). Meta-Analysis of the Associations of p-Cresyl Sulfate (PCS) and Indoxyl Sulfate (IS) with Cardiovascular Events and All-Cause Mortality in Patients with Chronic Renal Failure. PLoS ONE.

[40] De Faria Barros A., Borges N.A., Nakao L.S., Dolenga C.J., do Carmo F.L., de Carvalho Ferreira D., Stenvinkel P., Bergman P., Lindholm B., Mafra D. (2018). Effects of probiotic supplementation on inflammatory biomarkers and uremic toxins in non-dialysis chronic kidney patients: A double-blind, randomized, placebo-controlled trial. J. Funct. Foods.

[41] Karbowska M., Kaminski T.W., Marcinczyk N., Misztal T., Rusak T., Smyk L., Pawlak D. (2017). The Uremic Toxin Indoxyl Sulfate Accelerates Thrombotic Response after Vascular Injury in Animal Models. Toxins.

[42] Wang X., Chaudhry M.A., Nie Y., Xie Z., Shapiro J.I., Liu J. (2017). A Mouse 5/6th Nephrectomy Model That Induces Experimental Uremic Cardiomyopathy. JoVE.

[43] Leary S.L., Underwood W., Anthony R., Cartner S., Corey D., Grandin T., Greenacre C., Gwaltney-Brant S., McCrackin M.A., Meyer R. (2013). AVMA Guidelines for the Euthanasia of Animals: 2013 Edition.

[44] Muñoz D., Castillo H., Henriquez J.P., Marcellini S. (2018). Bone regeneration after traumatic skull injury in *Xenopus tropicalis*. Mech. Dev..

