# Supplementary material for: The Administration of the Synbiotic Lactobacillus bulgaricus 6c3 Strain, Inulin and Fructooligosaccharide Decreases the Concentrations of Indoxyl Sulfate and Kidney Damage in a Rat Model
Source: Toxins (Basel). 2021 Mar 7;13(3):192. doi: 10.3390/toxins13030192 (PMC7999732; doi:10.3390/toxins13030192)
Supplement: Supplementary file 1 [file toxins-13-00192-s001.pdf]

Supplementary Materials: The Administration of the Synbiotic *Lactobacillus bulgaricus* 6c3 Strain, Inulin and Fructooligosaccharide Decreases the Concentrations of Indoxyl Sulfate and Kidney Damage in a Rat Model

Alonso Jerez-Morales, José S. Merino, Sindy T. Díaz-Castillo, Carlos T. Smith, Jorge Fuentealba, Humberto Bernasconi, Gerson Echeverría and Apolinaria García-Cancino

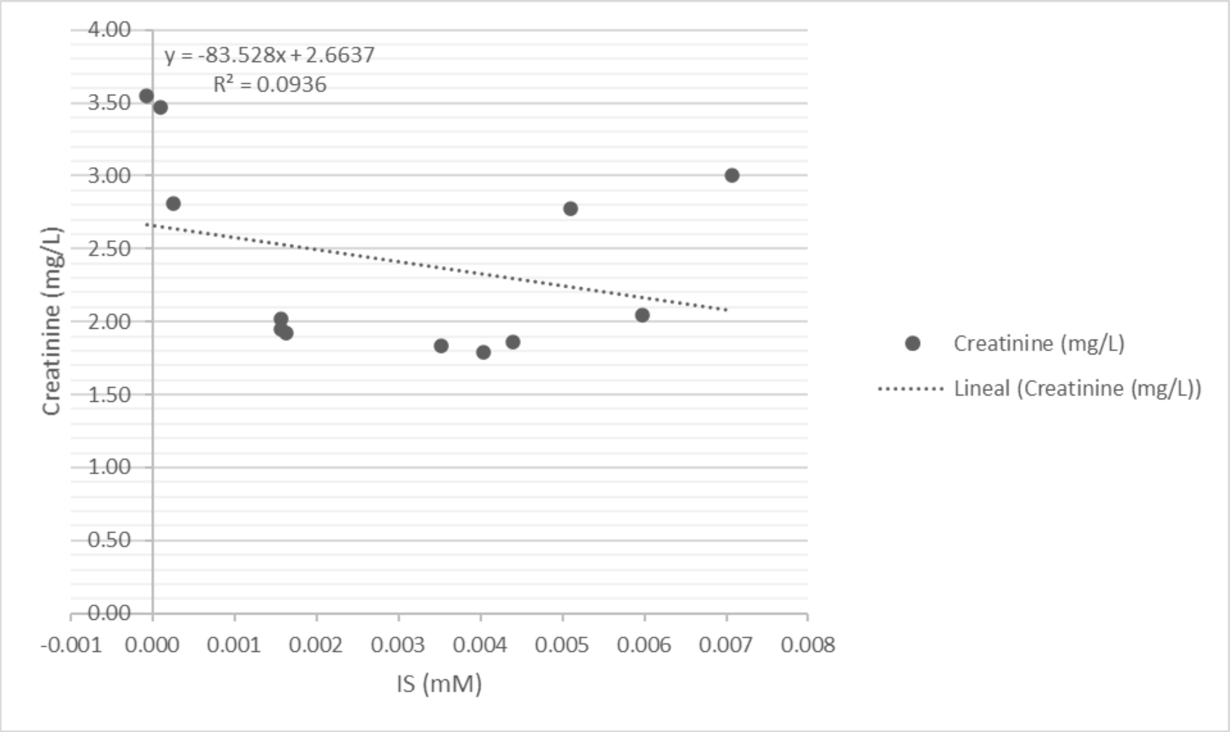

Figure S1. Correlation between creatinine and indoxyl sulfate (IS) levels of the control group.

 https://doi.org/10.3390/toxins13030192

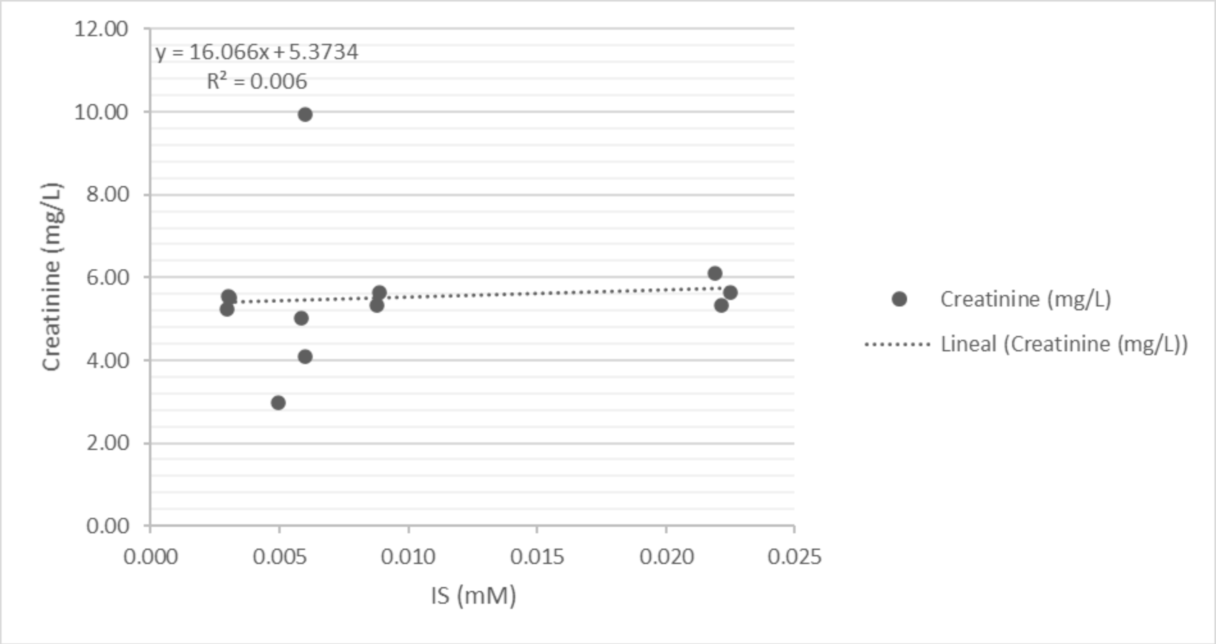

Figure S2. Correlation between creatinine and indoxyl sulfate (IS) levels of the Nef group.

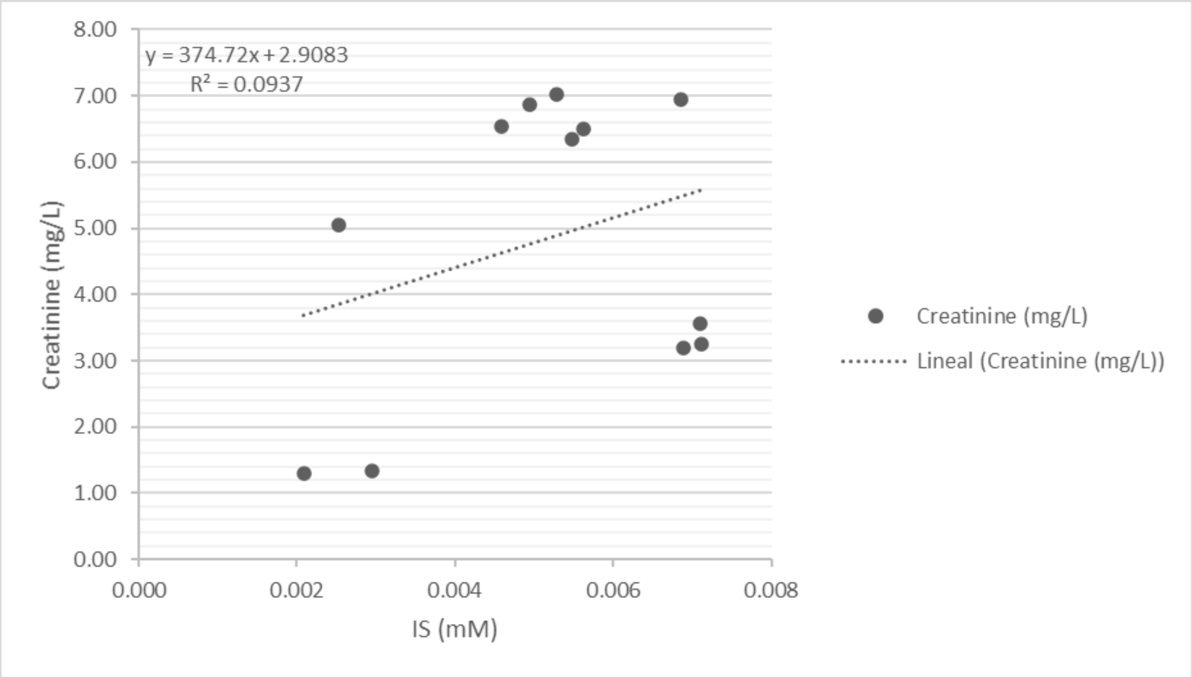

Figure S3. Correlation between creatinine and indoxyl sulfate (IS) levels of the Lac group.
